# What's Hot This Year in Infectious Diseases Clinical Science

**DOI:** 10.1093/cid/ciaf037

**Published:** 2025-02-01

**Authors:** Jeffrey A Freiberg, Patty W Wright

**Affiliations:** Division of Infectious Diseases, Department of Medicine, Vanderbilt University Medical Center, Nashville, Tennessee, USA; Vanderbilt Institute for Infection, Immunology, and Inflammation, Vanderbilt University Medical Center, Nashville, Tennessee, USA; Division of Infectious Diseases, Department of Medicine, Vanderbilt University Medical Center, Nashville, Tennessee, USA

## Abstract

Clinical science in the field of infectious diseases (ID) moved at a rapid pace in 2024. Among the highlights were multiple trials of new antibiotics and new approaches to prevent infections. Concerning trends, with outbreaks of dengue, measles, mpox, and highly pathogenic avian influenza A in 2024, demonstrate the importance of ID and the continued need for further advances through clinical science. This review highlights some of the most important trials and clinical trends in ID over the past year.

## Teaser

This review covers important public health notices and clinical trials covering a broad range of infectious diseases topics from October 2023 to September 2024. These updates were highlighted in the “What's Hot in ID Clinical Science” session at IDWeek 2024.

The infectious diseases (ID) community gathered in October 2024 for the annual IDWeek meeting, offering an opportunity to reflect on advancements in the field and challenges that remain. Summarizing a year's worth of progress into the “What's Hot in ID Clinical Science” session was not easy, as there were far more excellent clinical trials than could be covered in the allotted time frame. While there were many deserving studies, the following topics represent those that we choose to highlight at IDWeek 2024.

## NEW ANTIBIOTICS AND NEW USES FOR OLD ANTIBIOTICS

### Urinary Tract Infections

Due to antibiotic resistance and contraindications that sometimes limit the use of current first-line agents, there is room for new options in the treatment of urinary tract infections (UTIs). For the treatment of uncomplicated UTIs, the results of 2 phase 3 randomized controlled trials (RCTs), EAGLE-2 and EAGLE-3, were published in February 2024 [1]. These nearly identical trials tested gepotidacin, an oral, first-in-class triazaacenaphthylene antibiotic that inhibits DNA replication by binding 2 different topoisomerases at a binding site distinct from fluoroquinolones [2]. Geopotidacin was compared with nitrofurantoin by randomizing female patients over the age of 12 years with uncomplicated UTIs to receive 1 of the 2 drugs for 5 days. The primary outcome was therapeutic success, which was a combination of clinical and microbiologic resolution at day 10-13. Gepotidacin was noninferior to nitrofurantoin in terms of the primary outcome in EAGLE-2, while it was superior to nitrofurantoin in EAGLE-3. Notably, these differences were driven mostly by higher rates of microbiologic failure in the nitrofurantoin groups, as clinical success rates were relatively similar between the 2 arms. Gepotidacin has been submitted for Food and Drug Administration (FDA) review and a decision regarding its approval is expected in 2025 [3].

An older oral β-lactam antibiotic for uncomplicated UTI treatment, pivmecillinam, received FDA approval for use in the United States in spring 2024 [4]. Rates of resistance to pivmecillinam have remained very low despite several decades of widespread use internationally [5]. Pivmecillinam was already included as recommended treatment for uncomplicated cystitis in the 2011 Infectious Diseases Society of America/European Society of Clinical Microbiology and Infectious Diseases UTI guidelines, and with its FDA approval, it may well prove to be an important option for the treatment of uncomplicated UTIs in the United States [6].

The treatment of complicated UTIs, particularly those caused by extended-spectrum β-lactamase and carbapenemase-producing organisms, continues to be a major problem that often requires intravenous antibiotics. This year saw the publication of the CERTAIN-1 trial, which looked at intravenous cefepime-taniborbactam for the treatment of complicated UTIs [7]. In this study, patients were randomized 2:1 to receive either cefepime-taniborbactam or meropenem every 8 hours for 7 days, with the possibility of extending to 14 days in cases of bacteremia. In the study's microbiologic intent-to-treat population, patients who had a positive urine culture with an Enterobacterales or *Pseudomonas aeruginosa* isolate that was susceptible to both cefepime-taniborbactam and meropenem, cefepime-taniborbactam was superior to meropenem. The primary composite outcome of clinical success and microbiologic success at a 19–23-day test of cure was achieved in >70% of the patients in the cefepime-taniborbactam arm versus only 58% in the meropenem arm, although clinical success rates were similar (85.7% vs 81.1%, respectively) between the 2 arms. While cefepime-taniborbactam is an exciting prospect for treating resistant gram-negative infections, it is not yet FDA approved as its application was rejected in early 2024 due to concerns about the manufacturing process [8]. At this point, it is unclear when cefepime-taniborbactam may be available as an option for those difficult-to-treat infections.

### *Clostridioides difficile* Infections

Outside the urinary tract, *Clostridioides difficile* infections (CDI) remain another hot area for the development of new antibiotics and therapeutics. A novel bis-benzimidazole antibiotic, ridinilazole, was the subject of the Ri-CoDIFy 1 and 2 phase 3 RCTs published in 2024 [9]. Ridinilazole binds with high specificity to *C. difficile* DNA, which gives it a very narrow spectrum as it lacks activity against most other bacteria. This allows it to selectively kill *C. difficile* while preserving the rest of the gut microbiota. In these superiority trials, patients were randomized to receive 10 days of either ridinilazole or oral vancomycin. Although ridinilazole was not superior to vancomycin with regard to the primary outcome of sustained clinical responses and lack of recurrence within 30 days, it did have a significantly lower rate of recurrence, with only 8% of patients experiencing recurrence versus 17% for vancomycin. In addition, ridinilazole, compared with vancomycin, better preserved patients' microbiome diversity. Based on this RCT data, ridinazole is unlikely to receive FDA approval without further trials; however, it is encouraging to see the potential of this narrow-spectrum antibiotic for CDI.

### Syphilis

While not a new antibiotic, linezolid was tested for a new potential indication in the Trep-AB RCT published in April 2024 [10]. In that study, linezolid was tested for the treatment of early syphilis based on its success in in vitro and animal models. Patients received either 600 mg of oral linezolid daily for 5 days or a single dose of intramuscular benzathine penicillin G. Ultimately, the study was halted early due to safety concerns as only 70% of the patients in the linezolid arm met the primary end point of serologic response at 48 weeks, compared with 100% in the penicillin G group. The question remains whether more frequent dosing or longer courses of linezolid would be effective; at this time, however, linezolid does not appear to be a suitable alternative.

## NEW APPROACHES FOR ANTIMICROBIAL PROPHYLAXIS

### Ventilator-Associated Pneumonia

Ventilator-associated pneumonia (VAP) was the subject of 2 important prophylaxis studies published in recent years. The AMIKINHAL RCT randomized 850 patients in French intensive care units (ICUs) after ≥72 hours of ventilation to receive either daily inhaled amikacin or normal saline for 3 days [11]. The primary outcome was the development of VAP, as determined by a blinded committee using composite criteria including a requirement of microbiology data, in the first 28 days after randomization. Although the study showed a significant reduction in the rates of VAP, there was no reduction in antibiotic rates, length of stay, duration of mechanical ventilation, or mortality. This raises the possibility that inhaled amikacin might have been reducing the rates of VAP diagnosis in the study by sterilizing respiratory bacterial cultures without improving other important clinical outcomes.

Another RCT looking at the prevention of VAP, PROPHY-VAP, was published in 2024 and involved randomizing patients with acute brain injury within 48 hours of admission and within 12 hours of intubation to receive either a single dose of intravenous ceftriaxone or saline [12]. This study included 345 patients and was also conducted in French ICUs. It found not only a significant reduction in the primary outcome of the development of VAP between days 2 and 7 of intubation but also a significant improvement in other key clinical outcomes, including numbers of ventilator-free, antibiotic-free, and ICU- and hospital-free days and 28-day survival. Importantly, neither AMIKINHAL nor PROPHY-VAP showed a significant increase in the development of resistant organisms in either respiratory or rectal swab surveillance cultures.

### Prosthetic Joint Infections

Prosthetic joint infections are another infection associated with high morbidity and mortality rates. Current guidelines recommend a cephalosporin for surgical prophylaxis at the time of arthroplasty [13, 14]; however, this would not prevent infections due to methicillin-resistant *Staphylococcus aureus* (MRSA) or methicillin-resistant coagulase-negative *Staphylococcus*, among the most common causes of infections associated with implanted devices. Thus, there has been interest in the potential benefit of adding empiric vancomycin for surgical prophylaxis. In an RCT conducted among patients without a history of MRSA, whose results were published in late 2023 [15], the addition of vancomycin to cefazolin for surgical prophylaxis in arthroplasty did not reduce the risk of surgical site infections at 90 days. Vancomycin may still have some utility in patients at higher risk for MRSA, as the rate of MRSA carriage in this study was <1%. However, the results from this study suggest that it is not beneficial to broadly administer vancomycin for prophylaxis.

### Coronavirus Disease 2019

The prevention of coronavirus disease 2019 (COVID-19) infections continues to be an important area of focus, particularly among high-risk populations, such as immunocompromised patients, who are less likely to derive benefit from COVID-19 vaccines. The results from an RCT of nirmatrelvir-ritonavir published in 2024 suggest that nirmatrelvir-ritonavir does not have a role in postexposure prophylaxis against COVID-19 [16]. In that study, 2736 patients were randomized—within 96 hours of exposure to a household contact with COVID-19—to receive placebo or nirmatrelvir-ritonavir. There was no significant difference in the primary outcome, with the incidences of symptomatic severe acute respiratory syndrome coronavirus 2 (SARS-CoV-2) infection by day 14 being 3.9%, 2.6%, and 2.4%, respectively, in the placebo group, the group receiving 5 days of nirmatrelvir-ritonavir, and the group receiving 10 days of nirmatrelvir-ritonavir. The baseline seropositivity for SARS-CoV-2 was >90% in this study, which likely helps explain the low rates of household transmission seen and may have contributed to the lack of efficacy of nirmatrelvir-ritonavir.

## NEW STRATEGIES TO AVOID ANTIBIOTICS

### Probiotics

A double-blinded placebo-controlled study published in May 2024 randomized 174 women with histories of recurrent UTIs to 1 of 4 groups to look at the effect of commercially available probiotics, consisting of lactic acid bacteria and bifidobacteria, on UTI recurrence rates [17]. Patients received an oral and vaginal placebo, an oral probiotic with a vaginal placebo, a vaginal probiotic with an oral placebo, or both a vaginal and oral probiotic. The incidence of symptomatic UTI at 4 months was significantly lower in the groups receiving vaginal probiotics either with a placebo or with oral probiotics, compared with groups receiving placebo only or just an oral probiotic. This difference was still present at 12 months and translated to a significantly longer time to the first symptomatic UTI. This study provides the most definitive evidence yet that probiotics, and specifically vaginal probiotics, deserve consideration as a possible strategy to prevent UTIs and limit the need for antibiotic use.

### Acute Respiratory Tract Illnesses

Another RCT published in 2024 looked at the ability of nasal sprays to reduce the use of antibiotics for the treatment of acute respiratory illnesses [18]. In this study, 13 799 patients who were at high risk of adverse outcomes from respiratory infections or had a history of frequent respiratory tract infections were randomized to 1 of 4 arms: usual care, a gel-based nasal spray, a saline nasal spray, or a behavioral website. Those in the nasal spray groups were given instructions to use the nasal sprays at the first signs of an illness or after or during potential exposures. The primary outcome was the total number of self-reported days of respiratory tract illness in the previous 6 months, which was significantly reduced in both groups receiving nasal spray. Importantly, the number of courses of antibiotics, a key secondary outcome, was also significantly reduced in the nasal spray groups.

## COMBATING *S. aureus*

The treatment of *S. aureus* continues to be a hot area in ID clinical science as *S. aureus* is one of the leading bacterial causes of death both globally and in the United States [19]. Published in October 2023, the ERADICATE RCT was the first head-to-head phase 3 trial of an anti-MRSA antibiotic for bacteremia in 15 years [20]. In this study, 390 patients across 17 countries with complicated *S. aureus* bacteremia were randomized to receive either daptomycin or ceftobiprole in a double-blinded fashion. The primary outcome, treatment success at 70 days, was similar between the 2 arms and within the noninferiority margin; however, superiority of ceftobiprole was not demonstrated. Unfortunately, a subgroup analysis of outcomes in patients with MRSA was limited, as only about a quarter of the patients in the study had MRSA infections. Based on the results of this and other studies, ceftrobiprole was approved by the FDA in April 2024 [21].

The use of oral antibiotics for the treatment for *S. aureus* bacteremia also remains a topic of great interest. The SABATO noninferiority trial, published in 2024, randomized patients meeting a strict definition of uncomplicated *S. aureus* bacteremia to either complete 14 days of intravenous antibiotics or switch to oral therapy after 5–7 days to complete the remainder of a 14-day course [22]. The choice of antibiotics was dictated by the study protocol, based on antibiotic susceptibility testing data and patient allergies. As a testament to the difficulty in discerning which cases of *S. aureus* bacteremia are truly uncomplicated, >5000 patients were assessed for eligibility in order to enroll just 213 patients, only half of the original planned sample size. Despite this smaller sample size, the study met its noninferiority margin of 10% and showed a significant reduction in hospital length of stay in the group switched to oral therapy. This study adds to the growing literature suggesting that finishing a short treatment course with oral antimicrobials is safe in carefully selected patients with *S. aureus* bacteremia.

Despite data showing that universal ICU decolonization with daily chlorhexidine gluconate bathing and nasal mupirocin reduces the rates of MRSA infections in ICUs [23], there is hesitancy to adopt mupirocin decolonization given concerns about selecting for mupirocin resistance. Iodophor (providone-iodine) has been suggested as an acceptable alternative to mupirocin for nasal decolonization. In a large RCT, >800 000 patients across 137 US hospitals were randomized at the hospital level to receive either 5 days of twice daily iodophor nasal decolonization or 5 days of twice-daily mupirocin decolonization, in addition to receiving daily chlorhexidine gluconate bathing [24]. This study found that during the intervention period there was a significantly increased risk of recovering *S. aureus* in a clinical culture, attributable to a patient's ICU stay, in the patients who underwent nasal decolonization with iodophor. There were no differences, however, in the secondary outcome of all-cause bloodstream infections. Despite concerns for the potential development of mupirocin resistance, mupirocin is still the preferred option for *S. aureus* decolonization in ICUs, based on the results of this study.

## EMERGING AND REEMERGING THREATS

The ever-changing landscape of public health threats faced by the ID field frequently supports the idiom that “the only constant is change.” The last year was no exception as several new or reemerging threats rose to prominence.

### Dengue

The number of cases of dengue globally has risen since late 2023, with the Americas seeing approximately 3 times as many cases of dengue in 2024 as was seen in the previous year [25]. An increase has been seen in the United States mostly from travel-related cases, but some areas, such as Puerto Rico, are experiencing increased local transmission as well.

### Measles

Cases of measles have also been increasing both globally and in the United States, with >275 cases in the United States in 2024 [26, 27]. Most of these cases have been in unvaccinated individuals, with more two-thirds of cases occurring in children and nearly half of these cases requiring hospitalization. The majority are associated with international travel, as the incidence of measles outbreaks worldwide has increased due to a global decline in measles vaccination rates. The United States has also experienced decreases in vaccination rates, with the majority of states having kindergarten vaccination rates that fall below the 95% vaccination threshold required for herd immunity [27]. Given these local and global trends, it continues to be incredibly important to encourage measles-mumps-rubella vaccine uptake for all children, but especially in those traveling internationally. It is also important for physicians to be mindful of the symptoms of measles.

### Mpox

An outbreak of clade Ib mpox that began in the Democratic Republic of the Congo (DRC) in January 2023 has led to global concern, with the World Health Organization declaring the mpox outbreak a “public health emergency of international concern” in August 2024 after spread to several countries neighboring the DRC [28]. The first case of clade I mpox in the United States was reported in November 2024 [29]. As opposed to the notable 2022 global mpox outbreak, which was a clade II mpox virus, clade I viruses are more transmissible, more likely to be spread by means other than sexual contact, and more likely to cause severe infections [30]. Unfortunately, the National Institutes of Health announced that a trial conducted in partnership with the DRC's Institut National de Recherche Biomédicale, studying the use of the antiviral, tecovirimat for the clade I mpox outbreak in the DRC, was unsuccessful in reducing the duration of mpox lesions in children and adults [31]. This means that the smallpox and mpox vaccines given either before exposure or as postexposure prophylaxis are the best strategies available to treat clade I mpox at this time [32].

### H5N1

Undoubtedly the most rapidly changing situation with regard to public health and emerging infections in 2024 has been the ongoing highly pathogenic avian influenza (HPAI) A H5N1 outbreak. While H5 influenza viruses have been in circulation among wild birds and known to cause outbreaks among poultry for the last 15 years, infections in mammals have been sporadic and human cases very rare, with just 1 case in the United States before 2024 [33]. However, in March 2024, H5N1 was reported to be spreading among dairy herds in the United States. There were >900 confirmed cases in livestock across 17 states, with 10 states reporting a total of 66 cases of human infections in 2024 [33, 34]. The first severe human case of H5N1, due to exposure to backyard bird flocks, was reported in December 2024 [35]. Most cases, however, involve US farm workers and are attributed to poultry or dairy cow exposure, suggesting mammal-to-mammal spread. Also concerning is the finding of serologic evidence of a recent HPAI H5 infection in 7% of dairy workers in Michigan and Colorado [36]. This remains a situation that warrants close monitoring due to its pandemic potential.

## CONCLUSIONS

As new infectious threats continue to emerge and familiar threats continue to evolve, the challenges faced by the field of ID are never constant. The ability of the field to respond with new antimicrobials, new therapeutics, and new approaches provides encouragement for the ability of ID to meet these challenges. We look forward to seeing what advancements come through ID clinical research in 2025 and beyond.

## Note

***Financial support.*** This work is supported by the National Institute of Allergy and Infectious Diseases, National Institutes of Health (grant K08AI185310 to J. A. F.).
